# Localized amyloidoma of the chest wall: diagnostic and surgical perspectives

**DOI:** 10.1186/s44215-023-00045-2

**Published:** 2023-06-02

**Authors:** Toru Kawakami, Jun Atsumi, Kiyomi Shimoda, Miyako Hiramatsu, Teruaki Oka, Yuji Shiraishi

**Affiliations:** 1 Department of Thoracic Surgery, Fukujuji Hospital, 3-1-24 Matsuyama, Kiyose, Tokyo 204-8522 Japan; 2 Department of Pathology, Fukujuji Hospital, 3-1-24 Matsuyama, Kiyose, Tokyo 204-8522 Japan

**Keywords:** Amyloidoma, Light-chain amyloidosis, Chest wall tumor

## Abstract

**Background:**

An amyloidoma is defined as a solitary, localized, tumor-like deposit of amyloid in the absence of systemic amyloidosis. Amyloidomas occur most frequently in the bladder, followed by the lungs, trachea and bronchi, larynx and vocal cords, tonsils, conjunctivae, orbits, lymph nodes, gastrointestinal tract, and skin. It is extremely rare for an amyloidoma to present in the chest wall. Indeed, only 5 cases of chest wall amyloidoma have been reported. Moreover, all reported lesions were > 10 cm in size and involved the ribs. Herein, we report our experience with a patient who had a chest wall amyloidoma 3 cm in diameter without rib involvement.

**Case presentation:**

A 3.1 × 1.5-cm tumor situated in the fifth intercostal space of the chest wall was found in an 83-year-old male during a routine health checkup. Chest magnetic resonance imaging showed a mass of intermediate intensity on T1-weighted imaging and low intensity on T2-weighted imaging with heterogeneous contrast enhancement. The tumor was resected during video-assisted thoracoscopic surgery. No adhesions were observed between the tumor and the lung. The tumor was white, hard, elastic, and located in the extrapleural fat without bone involvement. The tumor was removed in a piecemeal fashion because the tumor was fragile and the surgical margin was unclear. A wide resection was achieved, including the intercostal muscle. Pathologic examination of the tumor using Dylon and Congo red staining confirmed amyloid deposition with tumor spread from the fat to the intercostal muscle and vascular walls but no pleural invasion. Without clear evidence of systemic amyloidosis, this patient was diagnosed with an amyloidoma of the chest wall. The postoperative course was uneventful, and he is doing well 1 year after surgery.

**Conclusion:**

Amyloidoma of the chest wall can present in various ways. An amyloidoma can be a small tumor, as in our patient, or the amyloidoma can be a large, destructive mass with rib involvement. Diagnosis of an amyloidoma should be kept in mind when patients present with chest wall tumors because an amyloidoma can be characterized by invasion within the chest wall independent of size.

## Background

“Amyloidosis” refers to a group of rare progressive disorders that cause deposition of amyloid, *an abnormal insoluble protein*, in various organs [1–3]. The heterogeneous pathogenic background of amyloidosis is classified into many subtypes, according to the chemical nature of the primary constituent amyloid protein [1, 2]. Light-chain amyloidosis (AL amyloidosis) is the most frequent type of systemic amyloidosis [1, 3]. The estimated prevalence of AL amyloidosis in Japan is 6.1 per million persons [3]. An amyloidoma is defined as a solitary, localized, tumor-like deposit of amyloid in the absence of systemic amyloidosis, and most amyloidomas are an uncommon manifestation of AL amyloidosis [1, 3]. It occurs most frequently in the bladder, followed by the lung, trachea and bronchi, larynx and vocal cords, tonsils, conjunctiva, orbits, lymph nodes, gastrointestinal tract, and skin [4]. It is extremely rare for an amyloidoma to be present in the chest wall. We report our experience with a patient who was diagnosed with a solitary localized amyloidoma of the chest wall during video-assisted thoracoscopic surgery (VATS).

## Case presentation

**Fig. 1:**
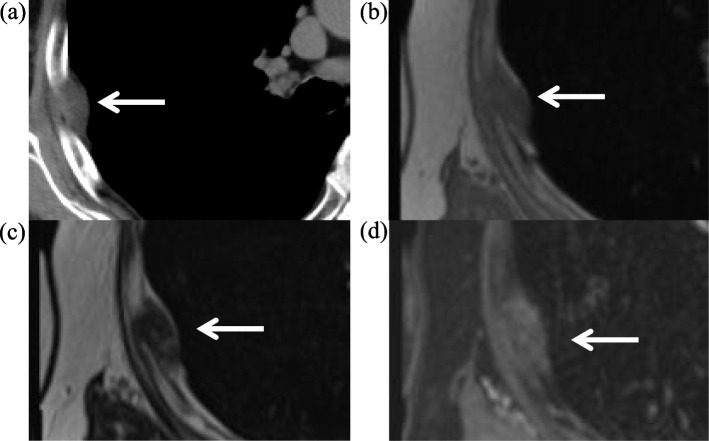
**a** Preoperative computed tomography. A soft-tissue mass measuring 3.1 × 1.5 cm (arrow) is seen in the right fifth intercostal space without bone involvement. **b** Preoperative T1-weighted MRI. The tumor (arrow) displays intermediate intensity on T1-weighted imaging. **c** Preoperative T2-weighted MRI. The tumor (arrow) displays low intensity on T2-weighted imaging. **d** Preoperative enhanced MRI. The tumor (arrow) displays heterogeneous enhancement

An 83-year-old man was referred to our hospital after an abnormal shadow was noted on chest radiography obtained as part of a routine health checkup. His medical history was significant for diabetes mellitus and a prior myocardial infarction, and his surgical history was significant for a cystostomy, performed after his urethra was torn during a traffic accident. He was a current smoker, with a smoking history of 10 cigarettes per day for 63 years. Chest computed tomography (CT) showed a mass in the right chest wall, measuring 3.1 × 1.5 cm, in the fifth intercostal space without bone involvement (Fig. 1a). Chest magnetic resonance imaging (MRI) showed a mass of intermediate intensity on T1-weighted imaging (Fig. 1b) and low intensity on T2-weighted imaging (Fig. 1c). The signal on T1- and T2-weighted imaging was almost the same intensity as that of skeletal muscle, and heterogeneous contrast enhancement was noted (Fig. 1d).

**Fig. 2:**
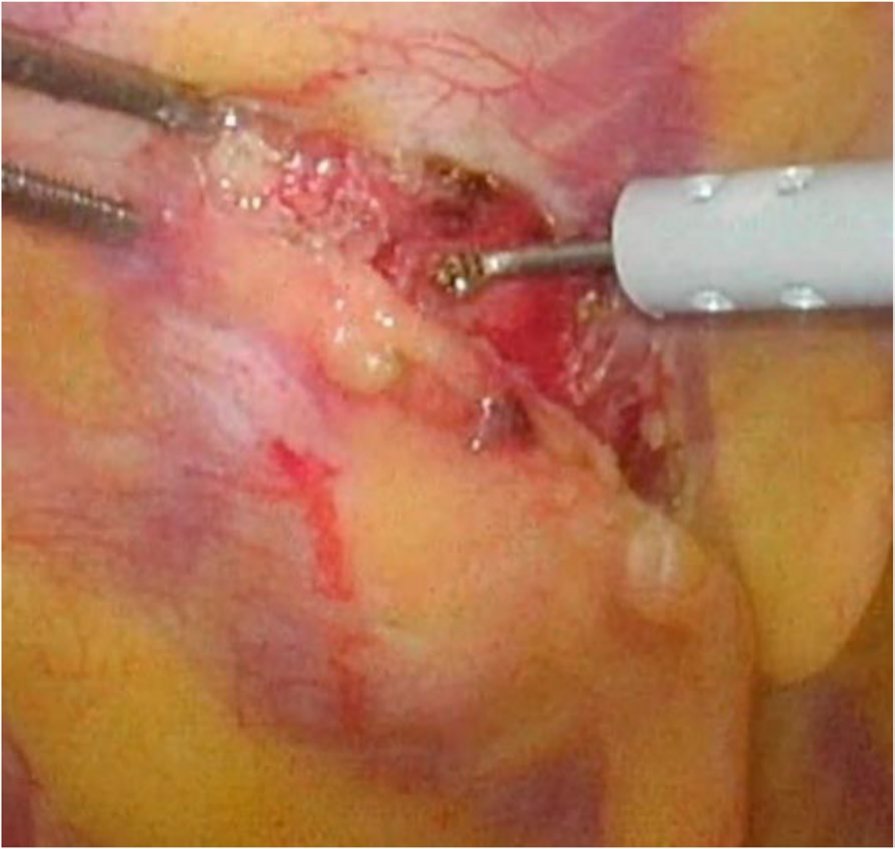
Intraoperative image. The tumor was white, hard, and elastic and located in the extrapleural fat. No adhesions were noted between the tumor and the lung

**Fig. 3:**
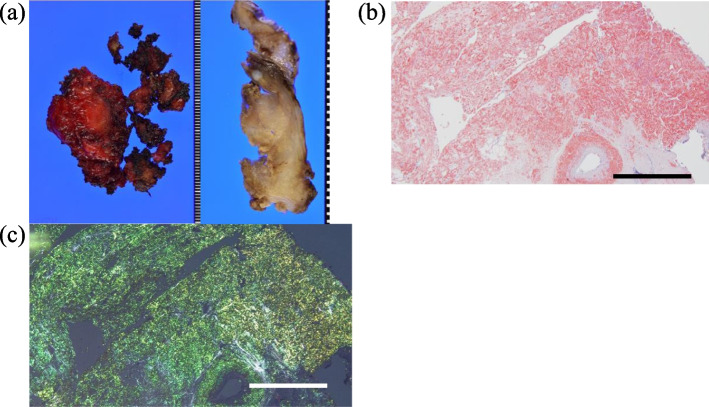
**a** Macroscopic pathologic findings. The tumor was resected in a piecemeal fashion. **b** Pathologic findings: Dylon staining. Amyloid deposition is shown by Dylon staining (scale bar, 500 μm). **c** Pathologic findings: Congo red staining. Amyloid deposition is shown by Congo red staining with apple-green birefringence on polarization microscopy (scale bar, 500 μm)

The tumor did not change in size for 7 months. The tumor was resected during VATS. No adhesions were observed between the tumor and the lung. The tumor was white, hard, and elastic and located in the extrapleural fat (Fig. 2). After incising the surrounding pleura, we attempted an en bloc resection. However, the tumor was fragile, and its surgical margin was unclear. We therefore removed the tumor in a piecemeal fashion (Fig. 3a), finally achieving a wide resection including the intercostal muscle. An intraoperative frozen pathologic diagnosis was not performed because the tumor was thought to be benign. Pathologic examination of the tumor using Dylon staining (Fig. 3b) and Congo red staining (Fig. 3c) indicated amyloid deposition. The tumor was diagnosed as a localized light-chain amyloidoma by proteomic analysis. Pathologic examination also showed that the tumor had spread from the fat to the intercostal muscle and the vascular walls, but no pleural invasion was observed.

The postoperative laboratory analysis, including serum and urine immunoglobulin levels, revealed no abnormalities. In addition, echocardiography and fluorodeoxyglucose-position emission tomography (FDG-PET) showed no clear evidence of systemic amyloidosis. Therefore, this patient was diagnosed with a solitary localized amyloidoma. His postoperative course was uneventful, and he is doing well 1 year after surgery.

## Discussion

It is extremely rare for an amyloidoma to occur in the chest wall. We have found 5 case reports of chest wall amyloidomas in the English-language literature [5–9]. All reported lesions were larger than 10 cm and involved the ribs; all were treated with extended resection of the chest wall, including rib resection. Amyloidoma of the chest wall is a very slow-growing tumor and typically causes few symptoms until it grows large enough to become destructive [5]. Large and destructive amyloidomas have been associated with severe dysfunction [5, 10]. Our patient had a chest wall tumor, which was an incidental finding during a routine health checkup. The definitive diagnosis was established postoperatively. This case represents the smallest amyloidoma reported to date and, unlike the lesions described in previous reports, did not have bone involvement. In addition, this is the first case in which a tumor was removed without extended chest wall resection. Nevertheless, the pathologic examination revealed that even such a small tumor can spread from the fat to the intercostal muscle and vascular walls. Despite being a benign tumor, a chest wall amyloidoma can be characterized by invasion within the chest wall. However, the number of reported patients with amyloidoma of the chest wall is limited, and further accumulation of cases is needed to make strong conclusions about the behavior of this tumor.

It is difficult to diagnose a chest wall amyloidoma based on radiologic findings because of the rarity of this tumor and the lack of reports describing the MRI or FDG-PET characteristics [5, 11]. This is the second report describing the MRI findings of a chest wall amyloidoma. The MRI findings in this patient were consistent with the MRI findings of other patients with chest wall amyloidomas: a heterogeneously enhancing mass of isointensity relative to skeletal muscle on T1-weighted imaging but relatively low intensity on T2-weighted imaging [5]. These findings are also consistent with the MRI findings of amyloidomas situated in other organs [12, 13]; however, these findings are not conclusive due to the limited number of cases. A histopathologic examination of the resected specimen is required to establish the correct diagnosis.

From a surgical point of view, it may be the case that amyloidoma is unlikely to invade the lung parenchyma. Only one reported patient with a chest wall amyloidoma had lung invasion [7]. The patient had an amyloidoma complicated by a plasmacytoma. The patient had radiotherapy prior to surgery; therefore, this was an atypical patient with an amyloidoma. In contrast, all reported amyloidoma cases had invasion within the chest wall. Furthermore, in our patient, the tumor was fragile, and the surgical margin was unclear, so we removed the tumor in a piecemeal fashion and achieved a wide resection. Considering these characteristics, we suggest that the tumor, as well as any involved surrounding tissues, should be excised when an amyloidoma is suspected.

With respect to patient prognosis, it is important to determine if a chest wall amyloidoma represents a solitary, localized tumor or a manifestation of systemic amyloidosis. In general, patients with an amyloidoma have marked FDG uptake on PET [3, 5, 11], which is consistent with the elevated glucose-analog utilization in tumors, especially pulmonary amyloidomas [11]. The postoperative whole-body FDG-PET findings for our patient showed no abnormal accumulation, which indicates no residual tumor or amyloidomas at other sites.

According to Mahmood et al., progression to systemic amyloidosis is exceptionally rare after resection of an amyloidoma, and the prognosis is generally excellent [4]. Resection of the localized tumor alone is usually adequate treatment [6, 7]. Therefore, our patient can expect a good prognosis. Amyloidoma of the chest wall can present in various ways: it can be a small tumor, as in our patient, or it can become a large, destructive mass with rib involvement. The diagnosis of amyloidoma should be kept in mind when a patient presents with a chest wall tumor, as an amyloidoma can be characterized by invasion within the chest wall independent of size.

## Data Availability

Not applicable.

## Abbreviations

AL amyloidosis: Light-chain amyloidosis
VATS: Video-assisted thoracoscopic surgery
CT: Computed tomography
MRI: Magnetic resonance imaging
FDG-PET: Fluorodeoxyglucose-positron emission tomography
